# Superfine Grinding of Oat Powder for Filtration-Free Oat Milk Production: Effects on Powder Properties, In Vitro Digestion, and Oat Milk Quality

**DOI:** 10.3390/foods15132320

**Published:** 2026-06-30

**Authors:** Se-Ho Jeong, Ui-Chan Jeong, Hafiz Muhammad Shahbaz, Ki-Min Lee, Si-Yeon Kim, Donghwa Chung, Dong-Un Lee

**Affiliations:** 1 Department of Food Science and Technology, Chung-Ang University, Anseong 17546, Republic of Korea; calvin0223@naver.com (S.-H.J.); no1chan@naver.com (U.-C.J.); dlrlarla123@naver.com (K.-M.L.); 2 Department of Nutrition and Health, College of Medicine and Health Sciences, United Arab Emirates University, Al Ain 15551, United Arab Emirates; shahbaz@uaeu.ac.ae; 3 Department of Food Science and Technology, Seoul Women’s University, Seoul 01797, Republic of Korea; asdfisiof1@naver.com; 4 Food Technology Major, Graduate School of International Agricultural Technology, Seoul National University, Pyeongchang 25354, Republic of Korea; 5 Institute of Food Industrialization, Institutes of Green Bio Science and Technology, Seoul National University, Pyeongchang 25354, Republic of Korea

**Keywords:** oat milk, oat powder, superfine grinding, filtration-free processing, β-glucan

## Abstract

Oat milk (OM) has gained popularity as a plant-based dairy alternative; however, conventional filtration-based production removes oat pulp, leading to β-glucan loss and processing waste. This study investigated the effects of particle size reduction by different grinding techniques on the powder properties and in vitro digestion characteristics of oat powder (OP), and it evaluated the applicability of superfine OP to filtration-free OM production. OP was prepared at three particle sizes: coarse, fine, and superfine, using a blender, ultra-centrifugal mill, and ball mill, respectively. Decreasing particle size improved hydration properties, including water absorption capacity, swelling capacity, and water solubility. During in vitro digestion, OP-superfine showed higher dialyzable protein fraction, β-glucan extractability, and digestion extract viscosity than OP-coarse, indicating an enhanced release of proteins and viscosity-contributing soluble components. When applied to OM, OP-superfine increased viscosity, Brix, turbidity, and suspension stability, while particle size had only a minor influence on pH. Sensory evaluation showed that OM prepared with OP-superfine had reduced grittiness and throat-feel intensity while maintaining relatively high sweetness. These findings suggest that superfine grinding is a promising strategy for producing filtration-free OM with improved digestion-related properties, physical stability, and sensory quality.

## 1. Introduction

Oats (*Avena sativa* L.) are nutritionally valuable cereal grains rich in protein, essential amino acids, minerals, vitamins, and dietary fiber, particularly β-glucan, which is a soluble dietary fiber associated with cholesterol-lowering and glycemic-modulating effects [1,2,3]. Owing to these nutritional and health-promoting properties, as well as environmental concerns, lactose intolerance, cow’s milk allergy, ethical considerations, and changing consumer preferences, oat milk (OM) has gained increasing popularity as a plant-based dairy alternative [4]. Among plant-based milk alternatives, OM has emerged as one of the most widely consumed products after almond milk [5].

Conventional OM production generally involves enzymatic treatment, homogenization, and filtration to improve texture and mouthfeel. However, filtration removes oat pulp, which contains valuable components such as β-glucan, thereby potentially reducing the health benefits of the final beverage [6,7]. In addition, the generation of oat pulp as a by-product raises concerns regarding processing efficiency and sustainability [6]. Therefore, developing strategies to reduce or eliminate the filtration step while maintaining acceptable physicochemical properties, physical stability, and sensory quality is important for improving OM production.

Particle size reduction is a key processing approach for modifying the physicochemical and functional properties of food powders. In cereal-based matrices, milling techniques such as ultra-centrifugal milling, which uses high-speed impact and shear, and ball milling, which relies on collision and friction, can disrupt the cell wall structures, increase the specific surface area, and enhance the release and hydration of functional components, including proteins and β-glucan [8,9]. In particular, superfine grinding, which produces micrometer-scale particles, may improve powder dispersibility, hydration behavior, and nutrient accessibility during digestion. These changes could also enhance the physical stability and sensory properties of whole-oat OM by reducing the perception of coarse particles without the need for filtration. However, limited information is currently available on how superfine grinding affects the properties of the resulting oat powder (OP) and its applicability to filtration-free OM production.

Therefore, this study aimed to investigate the effects of particle size reduction by different grinding techniques on the powder properties and in vitro digestion characteristics of OP, including protein digestibility, β-glucan extractability, and the viscosity of the in vitro digestion extract. In addition, the applicability of superfine OP to filtration-free OM production was evaluated by analyzing the physicochemical and stability properties, as well as the sensory attributes, of OM prepared with OP of different particle sizes.

## 2. Materials and Methods

### 2.1. Preparation of OP

Fresh oats (*Avena sativa* L.) from Gangjin-gun, Jeollanam-do, Republic of Korea, were used in this study. The oat samples were washed with distilled water and soaked for 10 min to raise the moisture content to approximately 20%. This soaking was followed by a 2 h tempering step to ensure uniform moisture distribution, which is critical for improving grinding efficiency and producing powders with consistent particle size. The tempered oats were dried in two stages: initially at 155 °C for 30 min and then at 33 °C for 12 h using a hot air dryer (SFC-203, Shinsaeng, Paju, Republic of Korea). The dried samples were ground into a coarse powder using a general blender. For fine powder, an ultra-centrifugal mill (Zm-200, Retsch, Haan, Germany) was used, and superfine powder was obtained using a ball mill (Pulverisette 5, Fritsch, Idar-Oberstein, Germany). The ultra-centrifugal mill operated at 6000 rpm with a 200 µm sieve, whereas the ball mill processed 200 mL of coarse OP with 2000 zirconium dioxide (5 mm) balls in a 500 mL grinding bowl at 350 rpm for 30 min. The coarse, fine, and superfine powders were denoted as OP-coarse, OP-fine, and OP-superfine, respectively.

### 2.2. Physical Properties of OP

#### 2.2.1. Particle Size Distribution

Particle size distribution was analyzed using a laser diffraction particle size analyzer (Mastersizer 3000, Malvern Panalytical Ltd., Malvern, UK) in dry dispersion mode, as described by Heo et al. [10]. The analyzer’s settings were as follows: particle absorption index of 0.1, dispersant refractive index of 1.00, laser obscuration of 1.45%, and particle refractive index of 1.53.

#### 2.2.2. Scanning Electron Microscopy (SEM)

Microstructures were observed using a scanning electron microscope (S-3400N, Hitachi High-Technologies Co., Tokyo, Japan). Samples were affixed to carbon tape on aluminum stubs and coated with platinum-lead (Pt-Pb) before observation at 150×, 500×, and 1000× magnifications.

#### 2.2.3. Color

Color values (L*, a*, b*) were measured using a color difference meter (CM-36dG, Konica Minolta, Tokyo, Japan) in the reflex mode. The calibration was performed using a standard black and white tile. The total color difference (ΔE*) was calculated by the following equation:

(1) $$\Delta E=\;\sqrt{{\left(\Delta L^{*}\right)}^{2}+{\left(\Delta a^{*}\right)}^{2}+{\left(\Delta b^{*}\right)}^{2}}$$

#### 2.2.4. Bulk Density, Tap Density, Carr Index, and Hausner Ratio

The bulk density and tap density of OP were determined using a graduated cylinder filled with 100 mL of OP. Bulk density was calculated as the weight of the powder (g) divided by the powder volume (100 mL). Tap density was measured after tapping the cylinder 100 times to remove gaps between particles. The Carr index (CI) and Hausner ratio (HR) were calculated using the following equations, respectively:

(2) $$Carr\;index\left(CI\right)\left(\%\right)=\frac{Tap\;density-Bulk\;density}{Tap\;density}\times 100$$

(3) $$Hausner\;ratio\left(HR\right)=\frac{Tap\;density}{Bulk\;density}$$

Flowability was evaluated using CI and HR with the following classification: CI < 10% or HR < 1.11 = excellent; CI 11–15% or HR 1.12–1.18 = good; CI 16–20% or HR 1.19–1.25 = fair; CI 21–25% or HR 1.26–1.34 = passable; CI 26–31% or HR 1.35–1.45 = poor; CI 32–37% or HR 1.46–1.59 = very poor; CI > 38% or HR >1.60 = extremely poor [11,12].

#### 2.2.5. Hydration Properties

Water absorption capacity (WAC) was measured according to the method described by Esposito et al. [13] with minor modification. WAC was determined by adding 0.5 g of OP to 10 mL of distilled water, stirring, and letting it stand for 18 h at room temperature. The suspension was centrifuged (3000× *g*, 20 min), the supernatant was decanted, and the residue was weighed. WAC was calculated as follows:

(4) $$WAC\left(g/g\right)=\frac{Weight\;of\;absorbed\;water\;(g)}{Weight\;of\;dry\;sample\;(g)}$$

Swelling capacity (SC) was measured according to the method described by Robertson et al. [14] with minor modification. SC was determined by adding 0.5 g of OP to 10 mL of distilled water, stirring, and letting it stand for 18 h at room temperature. The final volume was measured, and SC was calculated as follows:

(5) $$SC\left(mL/g\right)=\frac{Volume\;of\;wet\;sediment\;(mL)}{Weight\;of\;dry\;sample\;(g)}$$

Water solubility index (WSI) was measured according to the method described by Phat et al. [15] with minor modification. WSI was determined by dispersing 1 g of OP in 10 mL of distilled water, stirring for 1 h, and centrifuging (3500× *g*, 10 min). The supernatant was dried at 105 °C for 24 h and weighed. WSI was expressed as the ratio of dried supernatant solids to the initial dry sample weight (g/g) and calculated as follows:

(6) $$WSI\left(g/g\right)=\frac{Weight\;of\;dry\;supernatant\;(g)}{Weight\;of\;dry\;sample\;(g)}$$

### 2.3. In Vitro Digestion Properties

#### 2.3.1. In Vitro Digestion Model

The in vitro digestion procedure was based on validated gastrointestinal digestion models widely used for cereal and plant-based matrices, following the methods of Lee et al. [16] and Mäkelä et al. [17]. For the oral phase, 5 g of OP and 5 mL of saliva (pH 6.8) were stirred at 150 rpm for 5 min at 37 °C. For the stomach phase, 10 mL of gastric juice (pH 1.5) was added and stirred at 150 rpm for 30 min at 37 °C. For the small intestine phase, dialysis tubes (molecular weight cut-off, 50 kDa; Membrane Filtration Products, Inc., Seguin, TX, USA) filled with 5 mL of phosphate buffer (pH 7.0) were placed in the mixture along with 10 mL of duodenal juice and 5 mL of bile juice and stirred at 150 rpm for 90 min at 37 °C. Detailed compositions and enzyme activities of the simulated digestive fluids are provided in Table A1.

#### 2.3.2. Dialyzable Protein Fraction

Dialyzable protein fraction was measured in the phosphate buffer inside the dialysis tube (see Section 2.3.1) using the Bradford Protein Assay Kit (Takara Bio, San Jose, CA, USA), which measures the absorbance change from 465 to 595 nm when Coomassie dye binds to a protein.

#### 2.3.3. β-Glucan Extractability

β-glucan content was determined using a mixed-linkage β-glucan kit (Megazyme, Wicklow, Ireland) following AACC-approved method 32–23.01. β-glucan was decomposed into D-glucose using lichenase and β-glucosidase, and absorbance was measured at 510 nm. Extractability was calculated as the ratio of β-glucan content extracted during in vitro digestion to the total β-glucan content of the oats.

#### 2.3.4. Viscosity of In Vitro Digestion Extract

The viscosity of the in vitro digestion extract was measured using a Vibro Viscometer (SV-10, A&D Co., Tokyo, Japan) at 20 °C.

### 2.4. Preparation of OM

**Figure A1:**
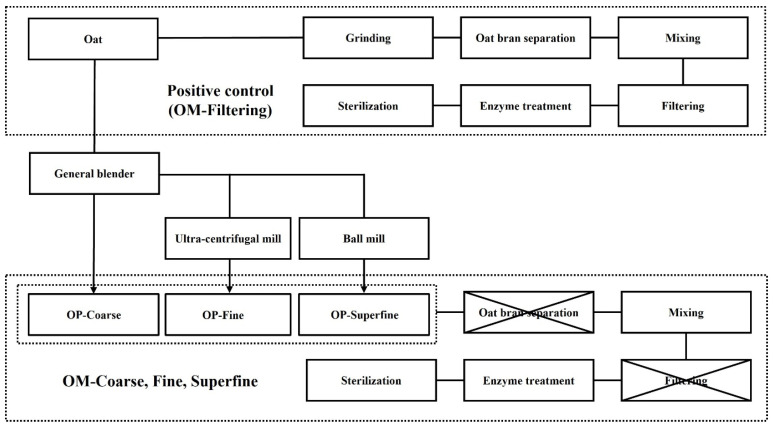
Schematic diagram of oat milk (OM) preparation using oat powders (OP) of different particle sizes.

OM samples were prepared following the previous method by Deswal et al. [18] with minor modifications. OP was mixed with distilled water in a 1:10 ratio (wt.%), stirred for 30 min, and heated at 65 °C for 30 min. α-amylase (BAN^®^ 480L, Novozymes, Bagsværd, Denmark; declared activity: 480 KNU/g) was added at 3 µL/g OP, and the mixture was incubated at 65 °C for 1 h with continuous stirring. The α-amylase was inactivated by heating at 121 °C, 1 bar for 10 min. For the filtered positive control, insoluble oat bran residues were removed by filtration after enzymatic treatment. In contrast, the experimental OM samples were prepared without a filtration step, and therefore retained the whole oat material, including bran-derived particles, in the final product. The samples were stored at 4 °C until required for analysis. A detailed schematic of OM preparation is provided in Figure A1.

### 2.5. Physicochemical Properties of OM

#### 2.5.1. Color

Color values (L*, a*, b*) were measured using a color difference meter (CM-36dG, Konica Minolta) in the transmittance mode. A standard black tile and deionized water were used to calibrate the instrument. ΔE* was calculated using the same equation in Section 2.2.2.

#### 2.5.2. pH, Viscosity, Brix, and Turbidity

The pH was measured using a pH meter (SevenEasy S20, Mettler-Toledo, Zürich, Switzerland). Viscosity was measured with a Vibro Viscometer (SV-10, A&D Co.). Brix was measured using a refractometer (Atago Co., Tokyo, Japan). Turbidity was measured as the optical density at 600 nm (OD_600_) using a spectrophotometer (Genesys 20, Thermo Fisher Scientific, Waltham, MA, USA).

#### 2.5.3. Suspension Stability

Suspension stability was measured following the previous method by Luo et al. [19] with minor modifications. OM samples were poured into 10 mL graduated cylinders and left at room temperature for 24 h. The volume of the supernatant was measured at 6 h intervals. Suspension stability was calculated using the following gravitational sediment ratio:

(7) $$Gravitational\;sediment\;ratio\left(\%\right)=\frac{Volume\;of\;supernatant\;(mL)}{Total\;volume\;of\;suspension\;mixture\;(mL)}\times 100$$

### 2.6. Sensory Evaluation

The sensory evaluation of OM was approved by the Chung-Ang University Institutional Review Board (No: 1041078-20230816-HR-225). Three parameters were analyzed: sweetness, mouthfeel grittiness, and throat-feel. Ten trained panelists evaluated the samples prepared in three independent batches. Each panelist evaluated each sample in triplicate using standard references for each attribute, and the average score of each panelist was used for statistical analysis. The descriptions of the references are provided in Table 1. The panelists evaluated each attribute using a 10 cm visual analog scale, which was anchored with “weak” on the left and “strong” on the right. This approach has been widely applied in sensory studies using trained panels for quantitative intensity evaluation [20]. Sensory scores were derived from the length (cm) marked on the line. Samples were coded using random three-digit numbers and presented in randomized order. Prior to evaluation, panelists were trained on the sensory attributes and use of the visual analog scale. Reference samples representing different intensity levels were provided to standardize interpretation of the scale among panelists.

### 2.7. Statistical Analysis

Data were expressed as the mean ± standard deviation (SD) of at least three measurements. Statistical analysis was performed using one-way analysis of variance (ANOVA), followed by Duncan’s multiple range test, using SPSS software version 20.0 (IBM Co., Armonk, NY, USA). Statistical significance was set at *p* < 0.05.

## 3. Results and Discussion

### 3.1. Physicochemical Properties of OP

#### 3.1.1. Particle Size Distribution of OP

The particle size distribution of OP samples produced using different milling techniques is presented in Table 2. Conventional blending (OP-coarse) produced the largest particles, with Dv_50_ and D_[4.3]_ values of 534.7 and 584.7 µm, respectively. This limited size reduction may be attributed to the relatively low macro-scale shearing and impact forces generated by blender blades, which were insufficient to extensively disrupt the fibrous oat matrix. In contrast, ultra-centrifugal milling (OP-fine) effectively reduced the particle size, resulting in Dv_50_ and D_[4.3]_ values of 106.7 and 166.3 µm, respectively. These values correspond to 5.0-fold and 3.5-fold reductions in Dv_50_ and D_[4.3]_, respectively, compared with OP-coarse. This enhanced size reduction can be explained by high-speed rotor-particle impact, wall friction, and shear forces, which fragment the oat matrix as particles pass through the sieve aperture [21]. However, OP-fine showed the highest span value (3.66), indicating a broader and less uniform particle size distribution.

The most drastic particle size reduction was achieved via ball milling (OP-superfine), which produced Dv_50_ and D_[4.3]_ values of 18.8 and 28.6 µm, respectively. The D_[4.3]_ value of OP-superfine was approximately 20.4 times smaller than that of OP-coarse and 5.8 times smaller than that of OP-fine. Ball milling operates through continuous multi-directional collisions and frictional forces between milling balls and powder particles [22]. These repeated compressive and shear stresses likely caused extensive disruption of the oat cell-wall network and starch–fiber complexes. Consequently, the specific surface area of OP-superfine reached 1685.7 m^2^/kg, which was 14.7 and 3.6 times higher than those of OP-coarse and OP-fine, respectively. This increase in specific surface area is consistent with the findings of Jiang et al. [23], who reported that reducing the particle size of onion peel powder increased its specific surface area. These results indicate that ball milling was the most effective technique for producing superfine OP, which may contribute to improved hydration and functional properties in oat-based food formulations.

#### 3.1.2. Microstructure and Visual Appearance of OP

**Figure 1:**
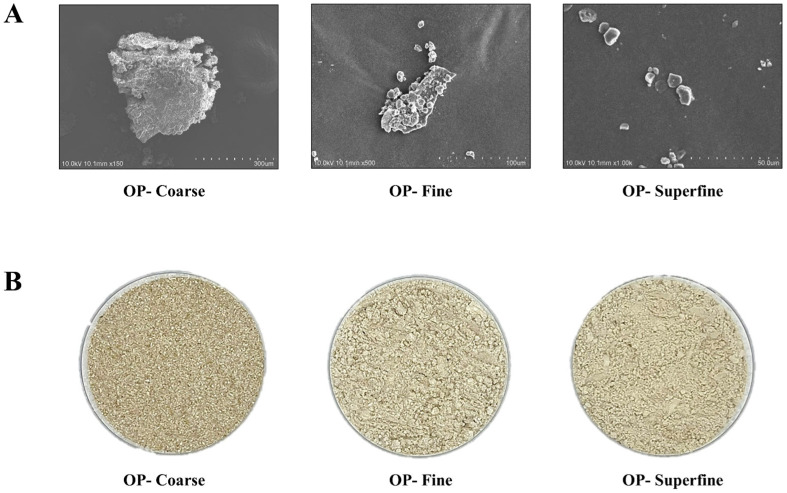
SEM micrographs (**A**) and visual appearance (**B**) of oat powders (OP) with different particle sizes. The SEM magnifications were 150× for OP-coarse, 500× for OP-fine, and 1000× for OP-superfine.

SEM micrographs revealed clear morphological differences among OP samples with different particle sizes (Figure 1A). OP-coarse consisted of relatively large and irregular particles, whereas OP-fine showed smaller fragmented particles. OP-superfine exhibited much finer particles with a more extensively disrupted structure, indicating effective particle size reduction by ball milling. These morphological observations were consistent with the particle size reduction results, in which OP-superfine showed the lowest D_[4.3]_ value and the highest specific surface area. The visual appearance of the powders also differed depending on particle size (Figure 1B). OP-superfine appeared lighter and more homogeneous than OP-coarse. These visual differences were further supported by the instrumental color measurements described in the following section.

#### 3.1.3. Color of OP

The CIELAB color values of OP samples with different particle sizes are shown in Table 3. The L* value, which represents lightness, increased significantly as particle size decreased. OP-superfine exhibited the highest L* value of 83.24, followed by OP-fine and OP-coarse, indicating that superfine grinding produced a lighter-colored powder. This result is consistent with the visual appearance shown in Figure 1B.

The a^*^ value decreased significantly with decreasing particle size, from 3.52 for OP-coarse to 1.75 for OP-superfine, indicating a reduction in redness. This decrease may be associated with the dilution of reddish–brown color characteristics as the oat matrix was more extensively fragmented into finer particles. In addition, the increased surface area of smaller particles may have enhanced light scattering, making the powder appear lighter and less reddish. The b* value, which represents yellowness, also differed significantly among samples. However, the value did not show a consistent particle size-dependent trend, suggesting that yellowness was not governed solely by particle size. The *ΔE* value increased with decreasing particle size with OP-superfine showing the highest *ΔE* value of 9.11 compared with OP-coarse. This indicates that particle size reduction substantially altered the overall color characteristics of OP.

The increase in L* after milling may be attributed to the greater specific surface area of smaller particles, which enhances light scattering and exposes the more internal structures of the oat matrix. Similar trends have been reported by Muttakin et al. [24] and Phat et al. [15], who observed increased brightness and lighter color in finely milled food powders. Jinapong et al. [25] also reported comparable changes in instant soymilk powders, supporting the relationship between reduced particle size and increased powder lightness.

#### 3.1.4. Density and Flowability

The bulk density, tap density, Carr index (CI), and Hausner ratio (HR) of OP samples are shown in Table 4. OP-coarse exhibited the highest bulk density of 0.51 g/mL, whereas OP-fine and OP-superfine showed lower values of 0.40 and 0.42 g/mL, respectively. The lower bulk density of the milled powders may be attributed to the formation of smaller and more irregular particles, which increased interparticle void spaces and reduced the packing efficiency under loose filling conditions. In addition, the increased specific surface area of finer particles may have enhanced cohesive interactions, further limiting efficient particle packing. In contrast, OP-superfine showed the highest tap density, suggesting that very fine particles could be rearranged into a more compact structure during tapping.

The CI and HR values increased significantly as particle size decreased, indicating reduced flowability. OP-coarse showed CI and HR values of 27.00 and 1.37, respectively, corresponding to poor flowability. OP-superfine exhibited the highest CI and HR values of 44.33 and 1.80, respectively, indicating extremely poor flowability. This reduction in flowability can be attributed to the increased specific surface area of smaller particles, which enhances interparticle friction, cohesive forces, and particle–particle interactions [11,12].

Although reduced flowability may be a disadvantage for dry powder handling, its practical impact may be limited in this study because the OP was intended for dispersion in water for OM preparation. Nevertheless, the reduced flowability of superfine powders should be considered in industrial applications, as it may affect powder handling, conveying efficiency, and storage performance. Moreover, the higher surface area and stronger particle–water interactions of finer powders may contribute to improved hydration behavior after reconstitution, as discussed in the following section.

#### 3.1.5. Hydration Properties of OP

The hydration properties of OP samples, including water absorption capacity (WAC), swelling capacity (SC), and water solubility index (WSI), are presented in Table 4. These properties indicate the ability of OP to interact with water during reconstitution and can influence the viscosity, dispersion behavior, and physical stability of OM.

All hydration properties were significantly affected by particle size. WAC increased from 1.15 g/g for OP-coarse to 1.66 g/g for OP-superfine, representing a 44% increase after ball milling. Similarly, SC increased from 3.27 to 4.80 mL/g, corresponding to a 46% increase. These increases can be attributed to the larger specific surface area and greater structural disruption of OP-superfine, which may provide more accessible sites for water binding and penetration into the oat matrix. The disruption of cell-wall structures may also expose hydrophilic components, including dietary fiber and proteins, thereby enhancing water uptake and swelling.

WSI also increased significantly as particle size decreased, from 0.05 g/g for OP-coarse to 0.1 g/g for OP-superfine. Although the absolute WS values were low, this increase suggests that superfine grinding promoted the release of water-soluble components from the oat matrix, which was likely through cell-wall disruption and the increased exposure of soluble carbohydrates and other hydrophilic constituents.

These findings are consistent with those of Qian et al. [1], who reported that reduced particle size improved the hydration properties of oat flour owing to increased specific surface area. The enhanced hydration properties of OP-superfine may contribute to improved dispersion and viscosity when applied to OM.

### 3.2. In Vitro Digestion Properties of OP

#### 3.2.1. Dialyzable Protein Fraction of OP

**Figure 2:**
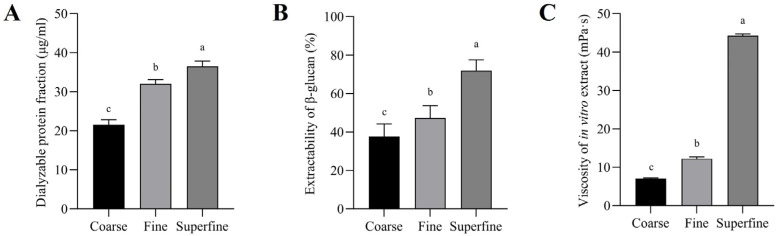
In vitro digestion properties of oat powders (OP) with different particle sizes; (**A**) dialyzable protein fraction, (**B**) β-glucan extractability, and (**C**) apparent viscosity of the in vitro digestion extract. Different letters indicate significant differences among samples (*p* < 0.05).

The protein digestibility of OP samples, expressed as a dialyzable protein fraction after in vitro digestion, is shown in Figure 2A. The protein fraction increased significantly as particle size decreased. OP-superfine showed the highest value of 36.5 µg/mL, which was approximately 70% higher than that of OP-coarse (21.5 µg/mL). This increase can be attributed to the structural changes induced by particle size reduction. Superfine grinding disrupts the oat cell-wall structure and loosens the starch–protein–β-glucan matrix [26], thereby increasing the exposure of proteins to digestive enzymes. The greater specific surface area of OP-superfine may also facilitate enzyme–substrate interactions, promoting protein hydrolysis and release during in vitro digestion. These findings agree with those of Junejo et al. [27], who reported that reducing the particle size of durum wheat bran increased protein extraction. Wang et al. [5] also demonstrated that particle size reduction in OM blends improved protein digestibility during in vitro digestion. Therefore, the higher protein fraction observed in OP-superfine indicates that superfine grinding improved the accessibility and release of oat proteins during digestion. It should be noted that the dialysis-based method estimates the dialyzable protein fraction rather than actual intestinal absorption. Thus, the present results should be interpreted as a comparative measure of protein availability among samples during in vitro digestion.

#### 3.2.2. β-Glucan Extractability of OP

The β-glucan extractability of OP samples during in vitro digestion ranged from 37.7% to 72.0%, depending on particle size (Figure 2B). OP-superfine showed the highest extractability, representing a 91% increase compared with OP-coarse. This enhancement can be attributed to the extensive disruption of the oat cell-wall structure and the increased specific surface area caused by superfine grinding. Oats generally contain approximately 3–7% β-glucan on a dry weight basis, and β-glucan is mainly located in the cell walls of the endosperm and aleurone tissues [2]. Therefore, structural disruption of the oat matrix can facilitate water penetration and promote the solubilization and release of β-glucan during digestion. In addition, the greater surface area of OP-superfine may increase the contact between the oat matrix and digestive fluids, thereby improving β-glucan extraction. These findings indicate that particle size reduction by ball milling enhanced the accessibility of β-glucan in OP during in vitro digestion. Similar observations have been reported in oat-based systems, where processing-induced changes in particle size and matrix structure affected the release and functionality of β-glucan [28]. Therefore, the higher β-glucan extractability observed in OP-superfine suggests that superfine grinding can improve the release of soluble dietary fiber from whole oat powder. However, β-glucan functionality is influenced not only by extractability but also by its molecular weight and structural integrity. Since the molecular weight of β-glucan was not evaluated in this study, the observed increase in extractability should be interpreted primarily as enhanced release during digestion rather than direct evidence of improved physiological functionality.

#### 3.2.3. Apparent Viscosity of In Vitro Digestion Extract of OP

The apparent viscosity of the in vitro digestion extract was significantly affected by particle size (Figure 2C). The digestion extract of OP-coarse showed the lowest viscosity of 7.07 mPa∙s. As the particle size decreased, the viscosity increased to 12.22 mPa∙s for OP-fine and 44.28 mPa∙s for OP-superfine, representing a more than six-fold increase compared with OP-coarse. The marked increase in viscosity for OP-superfine can be mainly attributed to the enhanced hydration properties and greater β-glucan extractability of OP-superfine (Table 4; Figure 2B). It should be noted that viscosity measurements were conducted at 20 °C for a comparative evaluation among samples rather than to simulate physiological conditions. In addition, the apparent viscosity measured using a vibro viscometer does not account for shear-dependent rheological behavior, which should be considered when interpreting the results.

The higher viscosity of the OP-superfine digestion extract may be associated with the increased extractability of soluble oat components, including β-glucan. This may be considered a favorable change in terms of β-glucan-related functionality, because the physiological functionality of cereal β-glucan is largely associated with its ability to form viscous solutions in the gastrointestinal tract [17]. In particular, increased digesta viscosity can slow nutrient diffusion and limit the transport of glucose and sterols to absorptive surfaces, thereby contributing to the glycemic- and cholesterol-modulating effects of β-glucan [17,29,30]. However, β-glucan functionality is also strongly influenced by its molecular weight and structural integrity. Since these parameters were not evaluated in this study, the observed increase in β-glucan extractability and digesta viscosity should be interpreted as evidence of enhanced release and solubilization during digestion rather than direct evidence of improved physiological functionality.

### 3.3. Physicochemical Properties of OM

#### 3.3.1. Appearance and Color of OM

**Figure 3:**
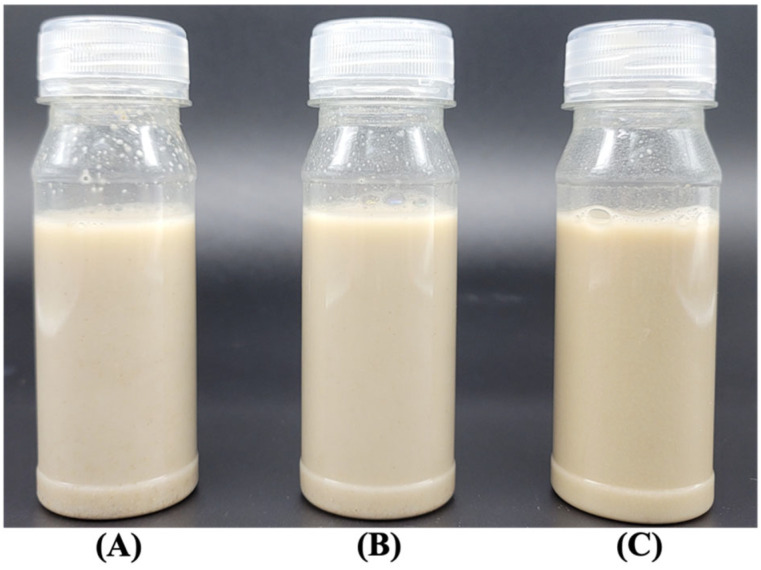
Visual appearance of oat milk (OM) prepared with oat powders (OP) of different particle sizes: (**A**) OM-coarse, (**B**) OM-fine, and (**C**) OM-superfine.

The visual appearance of OM was clearly affected by the particle size of OP (Figure 3). OM-coarse showed a relatively lighter and less turbid appearance, whereas OM-fine and OM-superfine appeared more opaque and yellowish. This visual difference was consistent with the instrumental color measurements presented in Table 5. The L* value decreased significantly as particle size decreased, from 61.36 for OM-coarse to 55.02 for OM-superfine, indicating reduced lightness in OM prepared with finer OP. In contrast, the a* and b* values increased with decreasing particle size, suggesting that OM-superfine had stronger reddish and yellowish color tones. The total color difference (*ΔE*) also increased with particle size reduction, indicating that the use of finer OP substantially altered the overall color characteristics of OM.

The reduced lightness and increased chromaticity of OM-superfine may be attributed to the greater dispersion of fine oat particles and soluble or suspended components in the aqueous phase. Smaller particles can remain more uniformly dispersed, increasing turbidity and producing a more opaque appearance. In addition, the increased surface area and structural disruption of OP-superfine may have promoted the release of soluble and suspended components from the oat matrix during OM preparation, which could further alter the optical properties of the beverage system. These results indicate that particle size reduction affected not only the color of OP itself but also the visual and optical properties of the resulting OM. Although thermal treatment during enzyme inactivation may have induced browning reactions to some extent, all OM samples were subjected to the same heating condition. Therefore, the differences in color among samples are considered to be more closely associated with particle-size-dependent dispersion and optical properties.

#### 3.3.2. pH, Viscosity, Brix, and Turbidity of OM

The pH, viscosity, Brix, and turbidity values of the OM samples are shown in Table 5. Particle size had only a minor influence on the pH of OM with values ranging narrowly from 6.40 to 6.49. In contrast, viscosity, Brix, and turbidity increased significantly as particle size decreased, reaching the highest values in OM-superfine.

The increase in viscosity can be attributed to the improved hydration properties of OP-superfine, as discussed in Section 3.1.5. The higher water absorption capacity, swelling capacity, and water solubility of OP-superfine may have promoted greater interactions between oat components and the aqueous phase, resulting in an increased viscosity of OM. In addition, the finer particles were more uniformly dispersed in the beverage matrix, which likely contributed to the higher turbidity of OM-superfine. Similar findings were reported by Cui et al. [31], who observed that reducing particle size increased viscosity and turbidity in chestnut-based beverages.

The increase in Brix with decreasing particle size was likely associated primarily with the α-amylase treatment used during OM preparation. The larger specific surface area and greater structural disruption of finer OP may have increased the accessibility of starch to α-amylase, thereby facilitating starch hydrolysis and promoting the release of soluble sugars, dextrins, and other soluble solids. Structural modifications induced by superfine grinding may have further enhanced the accessibility of starch and other oat components to enzymatic hydrolysis, although these changes were not directly evaluated in this study. Furthermore, superfine grinding may have enhanced the extraction of soluble components from the oat matrix during OM preparation. Therefore, the higher Brix value of OM-superfine likely reflects the combined effects of α-amylase-mediated hydrolysis and increased release of soluble components. Qian et al. [32] also reported that hydrolysis behavior was affected by particle size, supporting the particle size-dependent increase in Brix observed in this study. The thermal treatment (121 °C for 10 min) used to terminate enzymatic hydrolysis may have contributed to structural modifications such as starch gelatinization, protein denaturation, and polysaccharide interactions. However, since identical thermal treatment conditions were applied to all samples, the observed differences among treatments are considered to be primarily associated with particle size reduction.

#### 3.3.3. Suspension Stability of OM

**Figure 4:**
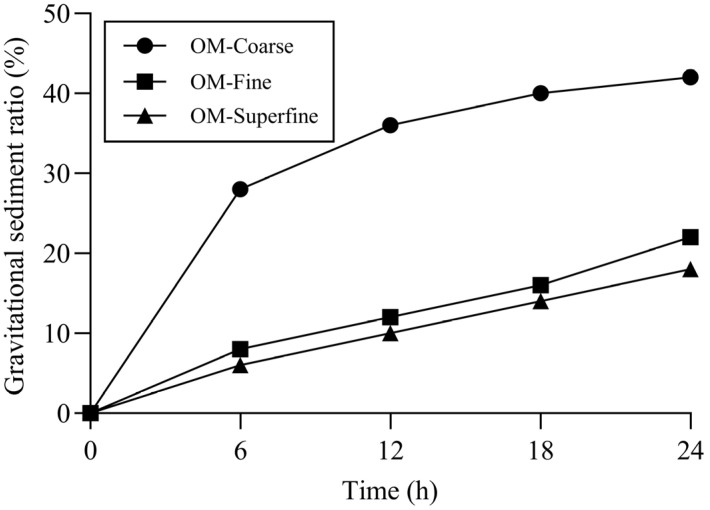
Changes in the gravitational sedimentation ratio of oat milk (OM) prepared with oat powders (OP) of different particle sizes during 24 h of static storage at room temperature.

The gravitational sedimentation ratios of OM samples during 24 h of static storage at room temperature are shown in Figure 4. OM-coarse exhibited rapid sedimentation, with sedimentation ratios of 28% after 6 h and 42% after 24 h, indicating poor suspension stability. In contrast, OM-fine and OM-superfine showed much lower sedimentation ratios of 8% and 6% after 6 h, and 22% and 18% after 24 h, respectively.

The improved suspension stability of OM-superfine can be attributed mainly to the reduced particle size and enhanced hydration properties of OP-superfine. Smaller particles have lower settling velocities and can remain dispersed for a longer period in the aqueous phase. In addition, the improved water absorption and swelling capacity of OP-superfine may have increased the viscosity of the continuous phase, thereby reducing particle sedimentation. These results are consistent with the viscosity and turbidity results described in Section 3.3.2, where OM-superfine showed the highest viscosity and turbidity values. Similar findings were reported by Luo et al. [19], who observed enhanced stability and more uniform dispersion in beverages containing finer sweet potato leaf powder particles. Overall, these results suggest that superfine grinding is effective in improving the suspension stability of filtration-free OM by reducing particle settling during storage.

In addition, the enhanced extractability of β-glucan may have also contributed to the improved suspension stability. As a soluble dietary fiber, β-glucan can increase the viscosity of the continuous phase and may help stabilize dispersed systems by reducing particle sedimentation. Similar effects have been reported in oat-based beverages, where β-glucan-containing formulations showed improved suspension stability, which was attributed to the formation of a more viscous continuous matrix [33].

### 3.4. Sensory Evaluation of OM

The results of the sensory evaluation of OM prepared with differently sized OP are shown in Table 6 with filtered OM used as the positive control. OM-superfine exhibited the highest sweetness score among the unfiltered samples, which is consistent with its higher Brix value. Notably, the sweetness score of OM-superfine was slightly higher than that of OM-filtered. This may be attributed to α-amylase-mediated hydrolysis during OM preparation, as the larger specific surface area of OP-superfine could facilitate starch breakdown and promote the release of soluble solids.

The grittiness score decreased from 7.47 for OM-coarse to 2.74 for OM-superfine, indicating that superfine grinding markedly reduced the perception of coarse particles in the mouth. This improvement can be attributed to the smaller particle size of OP-superfine (D[4,3] = 29 μm; Table 2). A similar trend was observed for throat-feel. The throat-feel score decreased from 4.92 for OM-coarse to 3.54 for OM-superfine, suggesting that particle size reduction contributed to a smoother swallowing sensation. However, OM-filtered showed lower grittiness and throat-feel scores than OM-superfine, indicating that filtration remains more effective in removing particles that contribute to mouthfeel and throat-feel sensations.

Overall, superfine grinding improved the sensory quality of filtration-free OM by reducing grittiness and throat-feel intensity while maintaining relatively high sweetness. These results suggest that OP-superfine can be used to produce filtration-free OM with improved sensory acceptability, although its sensory attributes were not identical to those of OM-filtered. Similarly, Ng et al. [34] reported that finer particles in blackcurrant squashes improved sensory attributes and overall acceptability. These findings suggest that superfine grinding may be a promising approach for reducing the need for filtration in OM production while improving the sensory properties of filtration-free OM. However, although the panel size (n = 10) was within the range commonly used for trained sensory evaluation, the sensory findings should be interpreted within the scope of this study.

## 4. Conclusions

This study demonstrates that superfine grinding can convert whole oat powder into a suitable ingredient for filtration-free OM production. Ball milling markedly reduced the particle size of OP and increased its specific surface area, leading to structural disruption of the oat matrix and improved hydration properties. These changes are important because enhanced water interaction and dispersion are essential for incorporating whole OP into beverage systems without filtration. Particle size reduction also improved the accessibility of oat components during in vitro digestion. OP-superfine showed higher protein digestibility, β-glucan extractability, and digestion extract viscosity than OP-coarse, indicating that disruption of the cell wall and starch–protein–β-glucan matrix promoted the release of digestible protein and viscosity-contributing soluble components. The increased viscosity is particularly meaningful because the physiological relevance of cereal β-glucan is closely associated with its ability to form viscous solutions in the gastrointestinal tract. However, the molecular weight and structural integrity of β-glucan, as well as other structural modifications potentially induced by ball milling, were not directly evaluated in this study. Therefore, further molecular and microstructural characterization is required to clarify the mechanisms underlying the observed improvements in digestibility, viscosity, and suspension stability as well as their physiological implications. When applied to OM, OP-superfine improved suspension stability and reduced particle-related sensory defects, including grittiness and throat-feel intensity. Although filtered OM still showed smoother sensory attributes, OP-superfine substantially narrowed the quality gap between unfiltered and filtered products. Overall, these findings highlight particle size control as a practical and sustainable strategy for developing whole-oat, filtration-free plant-based beverages with improved physical stability, sensory acceptability, and in vitro digestion properties.

## Figures and Tables

**Table 1:** Sensory attributes, definitions, and reference standards used for the evaluation of oat milk (OM) samples.

Sensory Attributes	Definition	Reference Standards
Sweetness	Characteristic taste of the sucrose solution	Weak: 0.1% sucrose in drinking water Strong: 10% sucrose in drinking water
Grittiness	The mouthfeel of the particles	Weak: milk Strong: perilla seed powder
Throat-feel	The feeling of passing through the throat	Weak: water Strong: yogurt

**Table 2:** Particle size distribution and specific surface area of oat powders (OP) with different particle sizes.

Samples	Dv_10_ (μm)	Dv_50_ (μm)	Dv_90_ (μm)	Span	Specific Surface Area (m^2^/kg)	D_[3,2]_ (μm)	D_[4,3]_ (μm)
OP-coarse	81.3 ± 2.3 ^a^	534.7 ± 1.7 ^a^	1116.7 ± 9.4 ^a^	1.93 ± 0.02 ^c^	114.8 ± 1.9 ^c^	174.3 ± 2.6 ^a^	584.7 ± 1.7 ^a^
OP-fine	16.7 ± 0.4 ^b^	106.7 ± 3.3 ^b^	407.3 ± 4.2 ^b^	3.66 ± 0.13 ^a^	473.4 ± 9.5 ^b^	42.3 ± 0.9 ^b^	166.3 ± 1.7 ^b^
OP-superfine	6.5 ± 0.0 ^c^	18.8 ± 0.1 ^c^	62.6 ± 0.5 ^c^	2.92 ± 0.01 ^b^	1685.7 ± 10.5 ^a^	11.9 ± 0.1 ^c^	28.6 ± 0.1 ^c^

Values are expressed as mean ± standard deviation (n = 3). Means with different letters are significantly different (*p* < 0.05).

**Table 3:** CIELAB color values of oat powders (OP) with different particle sizes.

Samples	L*	a*	b*	*ΔE*
OP-coarse	74.36 ± 0.05 ^c^	3.52 ± 0.01 ^a^	15.98 ± 0.08 ^a^	-
OP-fine	81.60 ± 0.06 ^b^	2.23 ± 0.01 ^b^	14.11 ± 0.02 ^c^	7.43
OP-superfine	83.24 ± 0.17 ^a^	1.75 ± 0.03 ^c^	14.42 ± 0.10 ^b^	9.11

Values are expressed as mean ± standard deviation (n = 3). Means with different letters are significantly different (*p* < 0.05).

**Table 4:** Density, flowability, and hydration properties of oat powders (OP) with different particle sizes.

Samples	Density		Flowability		Hydration Properties		
	Bulk Density (g/mL)	Tap Density (g/mL)	Carr Index (%)	Hausner Ratio	Water Absorption Capacity (g/g)	Swelling Capacity (mL/g)	Water Solubility (g/g)
OP-coarse	0.51 ± 0.01 ^a^	0.69 ± 0.00 ^b^	27.00 ± 0.82 ^c^	1.37 ± 0.02 ^c^	1.15 ± 0.03 ^b^	3.27 ± 0.19 ^c^	0.05 ± 0.01 ^c^
OP-fine	0.40 ± 0.00 ^c^	0.66 ± 0.00 ^c^	40.33 ± 0.47 ^b^	1.68 ± 0.01 ^b^	1.22 ± 0.03 ^b^	3.87 ± 0.09 ^b^	0.07 ± 0.00 ^b^
OP-superfine	0.42 ± 0.01 ^b^	0.76 ± 0.00 ^a^	44.33 ± 0.94 ^a^	1.80 ± 0.03 ^a^	1.66 ± 0.11 ^a^	4.80 ± 0.28 ^a^	0.10 ± 0.00 ^a^

Values are expressed as mean ± standard deviation (n = 3). Means with different letters are significantly different (*p* < 0.05).

**Table 5:** Physicochemical properties of oat milk (OM) prepared with oat powders (OP) of different particle sizes.

Samples	Color				pH	Viscosity	Brix	Turbidity
	*L* *	a*	b*	*ΔE*		(mPa∙s)	(%)	
OM-coarse	61.36 ± 0.26 ^a^	1.37 ± 0.11 ^b^	23.95 ± 0.22 ^c^	-	6.40 ± 0.03 ^b^	2.46 ± 0.01 ^c^	6.40 ± 0.12 ^b^	2.4 ± 0.0 ^c^
OM-fine	56.13 ± 0.63 ^b^	3.54 ± 0.28 ^a^	28.83 ± 0.44 ^b^	5.76	6.49 ± 0.03 ^a^	2.67 ± 0.01 ^b^	6.56 ± 0.25 ^b^	2.5 ± 0.0 ^b^
OM-superfine	55.02 ± 0.56 ^c^	3.82 ± 0.24 ^a^	29.41 ± 0.41 ^a^	6.88	6.47 ± 0.04 ^a^	2.94 ± 0.01 ^a^	6.96 ± 0.21 ^a^	2.7 ± 0.0 ^a^

Values are expressed as mean ± standard deviation (n = 3). Means with different letters are significantly different (*p* < 0.05).

**Table 6:** Sensory attributes of oat milk (OM) prepared with oat powders (OP) of different particle sizes.

Samples	Sweetness	Grittiness	Throat-Feel
OM-coarse	2.93 ± 1.20 ^b^	7.47 ± 1.45 ^a^	4.92 ± 1.57 ^a^
OM-fine	3.92 ± 1.45 ^ab^	5.74 ± 1.46 ^b^	4.28 ± 1.08 ^ab^
OM-superfine	4.17 ± 1.70 ^a^	2.74 ± 0.92 ^c^	3.54 ± 1.27 ^b^
OM-filtered (positive control)	3.29 ± 1.28 ^ab^	1.02 ± 0.80 ^d^	0.98 ± 0.67 ^c^

Values are expressed as mean ± standard deviation of panelist mean scores (n = 10), which were obtained from triplicate evaluations of samples prepared in three independent batches. Means with different letters are significantly different (*p* < 0.05).

## Data Availability

The original contributions presented in this study are included in the article. Further inquiries can be directed to the corresponding authors.

## Abbreviations

OP	Oat powder
OM	Oat milk
WAC	Water absorption capacity
SC	Swelling capacity
WSI	Water solubility index
CI	Carr index
HR	Hausner ratio

## Appendix A

**Table A1:** Constituents and concentrations of simulated digestive juices used in the *in vitro* model representing fed conditions.

	Saliva (Mouth)	Gastric Juice (Stomach)	Duodenal Juice (Small Intestine)	Bile Juice (Small Intestine)
Organic and inorganic components	1.7 mL NaCl (175.3 g/L) 8 mL urea (25 g/L) 15 mg uric acid	6.5 mL HCl (37 g/L) 18 mL CaCl_2_·2H_2_O (22.2 g/L) 1 g BSA	6.3 mL KCl (89.6 g/L) 9 mL CaCl_2_·2H_2_O (22.2 g/L) 1 g BSA	68.3 mL NaHCO_3_ (84.7 g/L) 10 mL CaCl_2_·2H_2_O (22.2 g/L) 1.8 g BSA 30 g bile
Enzymes	290 mg α-amylase ^(1)^ 25 mg mucin	2.5 g pepsin ^(2)^ 3 g mucin	9 g pancreatin ^(3)^ 1.5 g lipase ^(4)^	
pH	6.8 ± 0.2	1.5 ± 0.0	8.0 ± 0.2	7.0 ± 0.2

Total volume of the mixture was increased to 500 mL with distilled water. ^(1)^ α-Amylase from human saliva (Sigma-Aldrich, Saint Louis, MO, USA, A1031), activity 300–1500 U/mg protein. ^(2)^ Pepsin from porcine gastric mucosa (Sigma-Aldrich, P7000), activity ≥250 U/mg solid. ^(3)^ Pancreatin from porcine pancreas (Sigma-Aldrich, P1750), activity 4× USP specifications. ^(4)^ Lipase from porcine pancreas (Sigma-Aldrich, L3126), activity ≥125 U/mg protein.
